# Characterization of Iron and Organic Carbon Colloids in Boreal Rivers and Their Fate at High Salinity

**DOI:** 10.1029/2019JG005517

**Published:** 2020-04-07

**Authors:** Simon David Herzog, Luigi Gentile, Ulf Olsson, Per Persson, Emma Sofia Kritzberg

**Affiliations:** ^1^ Department of Biology/Aquatic Ecology Lund University Lund Sweden; ^2^ Department of Biology, MEMEG Unit Lund University Lund Sweden; ^3^ Department of Chemistry, Physical Chemistry Division Lund University Lund Sweden; ^4^ Centre for Environmental and Climate Research and Department of Biology Lund University Lund Sweden

**Keywords:** Fe speciation, natural colloids, size distribution, XAS, DLS

## Abstract

Riverine colloids are important carriers of macronutrients, trace metals, and pollutants into marine waters. The aim of the current study was to extend the understanding of iron (Fe) and organic carbon (OC) colloids in boreal rivers and their fate at higher salinities. X‐ray absorbance spectroscopy (XAS) and dynamic light scattering (DLS) were combined to explore Fe speciation and colloidal characteristics such as size and surface charge and how these are affected at increasing salinity. XAS confirmed the presence of two Fe phases in the river waters—Fe‐organic matter (OM) complexes and Fe(oxy)hydroxides. From DLS measurements on filtered and unfiltered samples, three particle size distributions were identified. The smallest particles (10–40 nm) were positively charged and suggested to consist of essentially bare Fe(oxy)hydroxide nanoparticles. The largest particles (300–900 nm) were dominated by Fe(oxy)hydroxides associated with chromophoric molecular matter. An intermediate size distribution (100–200 nm) with a negative surface charge was presumably dominated by OM and containing Fe‐OM complexes. Increasing the salinity resulted in a removal of the smallest distribution. Unexpectedly, both the intermediate and largest size distributions were still detected at high salinity. The collective results suggest that Fe(oxy)hydroxides and Fe‐OM complexes are both found across the wide size range studied and that colloidal size does not necessarily reflect either Fe speciation or stability toward salinity‐induced aggregation. The findings further demonstrate that also particles beyond the typically studied <0.45‐μm size range should be considered to fully understand the riverine transport and fate of macronutrients, trace metals, and pollutants.

## Key Points

- Three colloidal size distributions occurred in river water samples
- Positively charged Fe(oxy)hydroxide nanoparticles were removed at increasing salinity
- Larger size distributions were less affected by salinity

## 1 Introduction

In boreal streams and rivers, iron (Fe)‐ and organic carbon (OC)‐rich phases are important carriers of other elements and compounds (Filella et al., 1993; Hirst et al., 2017; Oni et al., 2013; Pokrovsky et al., 2006). These carriers span in size from molecules over colloidal to particulate phases. The colloids generally have a large specific surface area and high density of binding sites and therefore play a significant role in the transport and cycling of macronutrients, trace metals, and pollutants (Filella et al., 1993). The transport of colloids depends on their size, surface charge, and the chemical composition of the water (Hassellov & von der Kammer, 2008). Based on size separation (filtration, ultrafiltration, and flow field‐flow fractionation), a division between Fe‐rich and OC‐rich colloids has been made, where the former are predominantly found in the larger size fractions and the latter in the smaller fractions (Lyvén et al., 2003; Pokrovsky & Schott, 2002). It has been inferred that the larger Fe‐rich fraction consists of Fe(oxy)hydroxides (Hirst et al., 2017; Krachler et al., 2010; Neubauer et al., 2013; Stolpe & Hassellöv, 2007). Furthermore, elements associated to carrier phases distribute differently, where, for example, P, Al, Pb, V, W, Ti, Ge, Zr, Th, and light rare earth elements (REE) are found particularly in the larger and Fe‐rich fraction, while OC‐rich colloids in the smaller size range are important carriers of Mn, Co, Ni, Zn, Cu, Cd, Y, heavy REE, and also mononuclear Fe (Ingri et al., 2000; Lyvén et al., 2003; Pokrovsky & Schott, 2002).

When rivers discharge into estuaries, Fe displays a distinctly nonconservative behavior, with a major loss of Fe from the surface water following salinity‐induced aggregation and sedimentation (Boyle et al., 1977; Kritzberg et al., 2014; Pokrovsky et al., 2014; Sholkovitz et al., 1978). Assessment of Fe speciation by X‐ray absorption spectroscopy (XAS) has shown that Fe(oxy)hydroxides are highly susceptible to salinity‐induced aggregation and are selectively lost across salinity gradients (Herzog et al., 2017). The Fe that remains in suspension through the estuary has been suggested to be organically complexed mononuclear Fe (Krachler et al., 2010; Stolpe & Hassellöv, 2007). In line with that, the proportion of riverine Fe stable to salinity‐induced loss was found to be positively correlated to the contribution of organically complexed Fe (Herzog et al., 2020). From this it follows that the fate of riverine nutrients and trace elements in estuaries depend largely on the carrier phase to which they are associated (Pokrovsky et al., 2014; Pokrovsky & Schott, 2002). However, organically complexed Fe has been found also in salinity‐induced aggregates (Herzog et al., 2017). This implies that a division into two fractions—a small‐sized consisting of Fe‐organic matter (OM) complexes and a large‐sized consisting of Fe(oxy)hydroxides—with a distinctly different response to salinity may be overly simplistic. Indeed, the size distributions of Fe‐OM complexes and Fe(oxy)hydroxides are likely to be overlapping (Lead & Wilkinson, 2006; Pokrovsky & Schott, 2002). Therefore, studying Fe speciation or size distribution alone will not provide a complete understanding of what determines the fate of Fe and associated elements across salinity gradients.

In this study we combine methods that assess Fe speciation (XAS) with dynamic light scattering (DLS) that characterizes colloidal properties, to develop a more comprehensive understanding of Fe and OC colloids in boreal rivers and their response to estuarine salinity gradients. Several recent studies have demonstrated that Fe speciation of aqueous samples can be characterized with XAS, revealing information on oxidation state and neighboring atoms (Karlsson & Persson, 2012; Sjöstedt et al., 2013; Sundman et al., 2014) and confirming the prevalence of two Fe phases in boreal waters, Fe‐OM complexes and Fe(oxy)hydroxides. Size distributions of aqueous colloids can be determined with DLS and can in combination with measurements of surface charge provide information on reactivity and stability of natural colloids (Chekli, Phuntsho, Roy, & Shon, 2013; Palmer & von Wandruszka, 2001).

## 2 Materials and Methods

### 2.1 Sampling

Three rivers (Lyckeby, Mörrum, and Helge), located in southern Sweden and draining into the Baltic Sea, were chosen to provide a range of Fe and OC concentrations and variable Fe speciation, based on previous knowledge (Herzog et al., 2017). The catchments of all three rivers are dominated by coniferous forest, with minor agricultural and urban areas. Sampling was made just upstream the river mouth where there was no influence of seawater (Lyckeby: 56°11´55´´N, 15°39´47´´E; Mörrum: 56°11´21´´N, 14°45´00´´E; and Helge: 55°56´36´´N, 14°13´09´´E) in January 2016. Water samples were collected half a meter below the surface into acid‐washed polyethylene containers. Samples were filtered through a 150‐μm mesh to ensure homogeneous samples free of large detritus and stored cold and dark until return to the lab. Only acid‐washed material was used for sample handling. Oxygen (OxyGuard MkIII) and conductivity (HANNA HI 991300) were measured in the field. River samples were later analyzed for total Fe, OC, colloidal size distribution (DLS), zeta potential, Fe speciation (XAS), absorbance at 420 nm, and pH.

### 2.2 Filtration

DLS and surface charge (zeta potential) measurements were performed on both unfiltered and filtered samples, to obtain a higher resolution in the smaller size range. River samples were filtered through membrane filters (Millipore, Durapore) with pore sizes of 0.1, 0.22, and 0.45 μm. All filters were pre‐rinsed with Milli‐Q water, and the first 2 ml of the filtered samples were discarded. Total Fe, OC, DLS, and the zeta potential measurements were performed after filtration.

### 2.3 Artificial Seawater Mixing Experiments

To address how the colloidal distributions respond to increasing salinity, addition of artificial seawater to river water was made. Mixing experiments were initiated within 5 hr after sampling. The river waters were mixed with artificial seawater (6:1 vol:vol) to final salinities of 0, 1, 2, 7, and 25 g L^−1^ in 50‐ml Falcon tubes. This gradient was chosen because studies have shown that salinity‐induced aggregation takes place already at low salinities and most removal processes occur at salinities <15 (Stolpe & Hassellöv, 2010). Further, salinity 7 and 25 correspond to Baltic Proper and Kattegat. To achieve the desired salinities an artificial seawater stock solution was diluted with Milli‐Q water. This stock solution (245 g L^−1^) was produced using reagent grade salts following a standard protocol (Kester et al., 1967). After mixing, the samples were stored in the dark on a shaker for 12–24 hr to allow aggregation. Aggregates were then separated by centrifugation at 3,000 *g* for 8 hr at 4 °C. Total Fe, OC, pH, and salinity were measured in the supernatant after centrifugation, and the supernatant was also analyzed by DLS.

XAS analysis on salinity‐induced aggregates was performed on samples from River Lyckeby only. To collect sufficient amounts of salinity‐induced aggregates, a larger volume was required. A 5‐L water sample from River Lyckeby was adjusted to a salinity of 25 according to the protocol above. The larger volume required separation of the aggregated fraction in three sequential centrifugation steps. First, the samples were centrifuged at 4,271 *g* for 1.5 hr at 4 °C followed by a second and third step, both at 2,516 *g* for 15 min. The aggregates were then frozen and freeze‐dried and stored dark and dry until XAS analyses.

Finally, to link zeta potential to size distributions a 0.1‐μm filtered sample from River Lyckeby was mixed with artificial seawater to a salinity of 1 g L^−1^, and after 24 hr the sample was filtered again. Zeta potential, total Fe, OC, and pH were measured before and after the second filtration step.

### 2.4 XAS Sample Treatment and Data Collection and Analysis

To determine Fe speciation of river water by XAS analysis, a 1‐L sample was frozen as soon as possible and never more than 5 hr after collection. The samples were later freeze‐dried and stored dry in the dark until analyzed. Also, the freeze‐dried aggregates collected from River Lyckeby were analyzed for XAS. Fe K‐edge spectra were collected at beam line 4‐1 at the Stanford Synchrotron Radiation Lightsource (SSRL), USA. SSRL was running in a top‐up mode at 3.0‐GeV beam energy and at ca. 500‐mA ring current. The beam line was equipped with a Si[2 2 0] double crystal monochromator. Three consecutive ion chambers, to monitor the transmitted beam, and one passivated implanted planar silicon (PIPS) detector for fluorescence measurements were used. To reduce higher‐order harmonics the monochromator at 4‐1 was detuned (50%). K‐edge spectra were collected in a k range up to 14 Å^−1^ in a fluorescence mode. To reduce unwanted scattering and fluorescence contribution, a Mn filter and Soller slits were used. Samples were mounted in a liquid nitrogen cryostat for measurements (ca. 80 K), to prevent beam‐induced damage. The samples were aligned at 45° with respect to the incident beam. Depending on the Fe concentration, two to six scans were recorded for each sample. Simultaneously, the spectrum of an Fe reference foil was recorded, to allow energy calibration.

The data analysis was performed following the procedure in Herzog et al. (2017). In short, scans were energy calibrated and averaged using Sixpack (Webb, 2005). The same program was used to qualitatively compare the X‐ray absorption near edge structure (XANES) regions, both the normalized XANES spectra and the first derivative XANES spectra.

Oxidation state analysis from pre‐edge information was performed according to Wilke et al. (2001). The data pretreatment was accomplished using the XANES dactyloscope software (Klementiev, 2002), and the baseline corrected pre‐edge was fitted with two pseudo‐Voigt functions (50% Gaussian and 50% Lorentzian) using Fityk (Wojdyr, 2010). This yielded the integrated pre‐edge intensity and the pre‐edge centroid energy.

The averaged EXAFS spectra were analyzed according to a shell‐by‐shell fit procedure using Viper (Klementev, 2001). The background was removed from the normalized spectra by applying a smoothing spline function, and these spectra were k^3^‐weighted and subsequently Fourier transformed (FT) using Bessel window function. The spectra were fit in k‐space with theoretical phase and amplitude functions calculated by the ab initio code FEFF7 (Zabinsky et al., 1995). For the FEFF calculation the input structures of goethite (Oʼday et al., 2004) and trisoxalatoiron(III) complex (Persson & Axe, 2005) were selected, since they contained the relevant scattering paths (short Fe‐Fe, long Fe‐Fe, Fe‐C, and Fe‐C/O), which are needed to fit contributions from Fe(oxy)hydroxides and Fe‐OM. The amplitude reduction factor (S_0_^2^) was set to 0.80; the threshold energy (ΔE_0_) was varied but correlated so that it was identical for all shells. By correlating the coordination numbers and fixing the Debye‐Waller factors (σ^2^) to values found in the literature (Maillot et al., 2011; Persson & Axe, 2005), we restricted the number of free variables. To qualitatively differentiate between different backscatters, the wavelet transform (WT) method, using the Igor Pro script by Funke et al. (2005), was applied (Karlsson & Persson, 2010).

### 2.5 Dynamic Light Scattering and Electrophoretic Mobility Measurements

The Zetasizer Nano ZS instrument from Malvern Instruments, Ltd., Worcestershire, UK, was used for DLS measurements at θ = 173° in addition to some electrophoretic mobility measurements. The goniometer system was equipped with a 4‐mW He‐Ne laser with an automatic laser attenuator, and the detection unit comprised an avalanche photodiode. The temperature was set to 25 °C. Three consecutive DLS measurements were performed on the same solutions. The solutions were filled in disposable folded capillary cells, and the measurements were performed at a fixed scattering angle of 173° using a laser interferometric technique (laser Doppler electrophoresis), which enabled the determination of the electrophoretic mobility. In such an experiment, an electric field is applied to a dispersion of charged particles that move with a velocity (v = |v̅|), and the Doppler‐shifted frequency of the incident laser beam caused by these moving particles is monitored. The velocity of a particle with radius R moving in an applied electric field, E = |E̅|, is v = u_e_E, where u_e_ is the electrophoretic mobility. The zeta potential, ζ, was calculated from the Helmholtz‐Smoluchowski equation (Evans & Wennerström, 1999)

(1) $$\zeta =\frac{\eta }{\varepsilon_{0}\varepsilon_{r}}u_{e},$$

where *ε*_*r*_ is the dielectric constant of the medium, *ε*_0_ is the permittivity of vacuum, and *η* denotes the solvent viscosity. The presented electrophoretic mobility values are the averages of three consecutive measurements.

### 2.6 Standard Analytical Methods

Total Fe was determined with an ICP‐OES Optima 8300 (Perkin Elmer). These samples were acidified (1% vol, HNO3) 24 hr before measurement. Blanks (Milli‐Q water with 1% HNO3) and standards (Fe pure standard, Perkin Elmer) were included at the beginning and the end of each run. OC was analyzed by high temperature catalytic oxidation on a Shimadzu TOC V‐CPN analyzer, using the Nonpurgeable Organic Carbon (NPOC) mode on HCl‐acidified samples (pH < 2). Blanks and standards were included in each run, and a four‐point standard curve was used for calibration. pH was measured with a 913 pH Meter (Metrohm), salinity with an inoLab Cond 730 WTW, and absorbance at 420 nm using a Beckman Coulter DU‐800 spectrophotometer.

## 3 Results and Discussion

### 3.1 Characterization of the River Waters

Fe and OC concentrations in these boreal rivers are generally high (Kritzberg et al., 2014), but these samples taken at low discharge in winter displayed relatively low concentrations (Table 1), probably as a result of limited flow through organic soil layers and more input from groundwater (Ekström et al., 2016). The high concentrations of Fe and OC in relation to other elements that may contribute to colloidal phases support their role as major carriers and indicate that they are dominating the colloidal characteristics studied here. For instance, the Fe concentrations were 13–26 and 6–8 times higher than those of manganese and aluminum, respectively. Silica and calcium were the major chemical elements but prevail as dissolved species under these chemical conditions (Dahlqvist et al., 2004; Stumm & Morgan, 2012). Further, based on previous XAS studies on the same river systems, the fraction of clay minerals in the suspended fraction was insignificant (Herzog et al., 2020).

**Table 1:** Water Chemical Variables in the Three Rivers at the Time of Sampling, and the Relative Loss of Fe, OC, and Absorbance by Filtration Through Filters With a Pore Size of 0.45, 0.2, and 0.1 μm, Respectively

				Fe lost by filtration (%)				OC lost by filtration^a^ (%)				Abs lost by filtration (%)
River	pH	Conductivity (μS cm^−1^)	Fe (μM)	0.45 μm	0.2 μm	0.1 μm	OC (mM)	0.45 μm	0.2 μm	0.1 μm	Abs_420_	0.1 μm
River Lyckeby	6.6	94.7	19.1	23	29	35	1.90	5	5	4	0.092	46
River Mörrum	7.0	106.2	4.0	62	62	62	0.87	5	7	5	0.021	47
River Helge	7.1	147.5	21.9	24	26	29	1.23	3	5	2	0.076	47

a The differences in OC loss between the filter sizes are within the error of the measurements and not significantly different.

**Figure 1:**
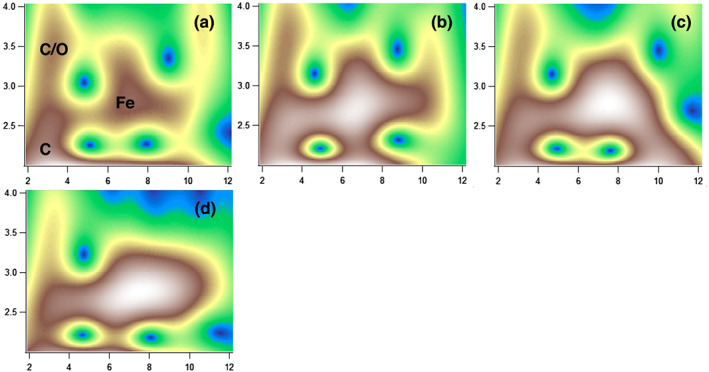
High‐resolution WT modulus (η = 8, σ = 1) of EXAFS data of River Lyckeby (a), River Mörrum (b), River Helge (c), and the aggregated fraction of Lyckeby at salinity 25 (d). The samples are plotted as a function of k (Å^−1^) on the *x*‐axis and R (Å) on the *y*‐axis.

The WT plots illustrate that the contribution of the two Fe phases varied among the rivers. The features representing Fe‐OM complexes (indicated as C and C/O in Figure 1a) were similar among the river samples, while the feature representing Fe(oxy)hydroxide (Fe in Figure 1a) was more distinct for Helge and Mörrum than for Lyckeby, indicating a higher contribution of Fe‐OM complexes in River Lyckeby. This was further confirmed by the coordination numbers (CN, contribution of a path to the samples) obtained from the extended X‐ray absorbance fine structure (EXAFS) fitting; that is, all samples contained significant, but variable, proportions of Fe‐OM complexes and Fe(oxy)hydroxides (Table 2). The CN_Fe‐C_/CN_Fe‐Fe_ ratios indicate a high contribution of Fe‐OM complexes in River Lyckeby and a more even contribution of the phases in River Helge and Mörrum. The significant contribution of both Fe(oxy)hydroxide and Fe‐OM complexes in all samples goes along with previous studies on similar systems (Herzog et al., 2017; Sundman et al., 2014) and agrees with the existence of two colloidal carrier phases in boreal river water, as proposed from size distribution and ultrafiltration studies (Andersson et al., 2006; Ingri et al., 2000; Pokrovsky & Schott, 2002).

**Table 2:** k^3^‐Weighted Fe K‐Edge EXAFS Fits for River Samples and the Aggregated Fraction From River Lyckeby

Site	Fe‐O (SS)			Fe‐Fe (SS)		Fe‐Fe (SS)		Fe‐C (SS)		Fe‐C/O (MS)^a^			Ratio^b^
	CN	R (Å)	σ^2^	CN	R (Å)	CN	R (Å)	CN	R (Å)	CN	R (Å)	∆E^0^	Fe‐C/Fe‐Fe
River Lyckeby	6.2	2.01	0.0011	0.9	3.08	0.5	3.46	2.4	3.03	4.8	4.17	3.7	2.6
River Mörrum	6.3	2.01	0.0014	2.1	3.09	1.0	3.40	2.5	2.92	5.1	4.20	3.6	1.2
River Helge	5.9	2.00	0.0010	1.6	3.05	1.0	3.50	2.1	3.05	4.2	4.07	2.9	1.3
Lyckeby Aggregation	5.7	1.98	0.0011	2.4	3.07	0.9	3.39	1.3	2.88	2.6	4.16	1.5	0.5

*Note*. Abbreviations were used for coordination number (CN), bond distance (R), and Debye‐Waller factor (σ^2^).

a Fe‐C/O is correlated to CN (Fe‐C) × 2. Also, the Debye‐Waller factor of Fe‐C/O was correlated to σ^2^ (Fe‐C) × 2. The σ^2^ for both Fe‐Fe was 0.01, adapted from Maillot et al. (2011), and for Fe‐C it was 0.0075 from Sundman et al. (2014).

b Ratio between the CN of the first Fe‐C path and the Fe‐Fe path.

**Figure 2:**
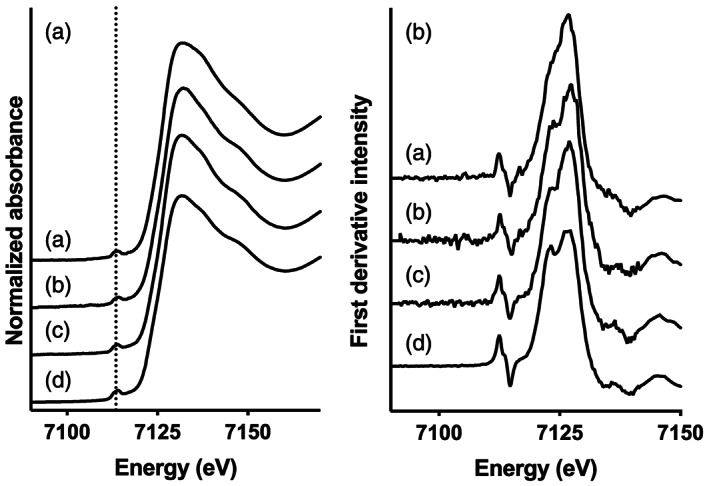
Normalized XANES spectra (a) and the corresponding first derivatives (b) of the river water samples: Ri ver Lyckeby (a), River Mörrum (b), River Helge (c), and the aggregated fraction from Lyckeby after increasing salinity (d).

The position of the pre and main Fe K‐edge indicated a predominance of Fe(III) in all samples (Figure 2), in agreement with previous studies on river mouth samples (Herzog et al., 2020). The double peak detected in the first derivative of the XANES spectra for Rivers Helge and Mörrum (Figure 2b) suggested a strong contribution from Fe(oxy)hydroxides (Sundman et al., 2014). For River Lyckeby this feature was less distinct, and the presence of a single peak indicated a dominance of amorphous Fe(oxy)hydroxides and of Fe‐OM complexes, which corroborated the results obtained from the EXAFS fits and WT analysis. A detailed description of the XAS analysis and results can be found in the supporting information.

**Figure 3:**
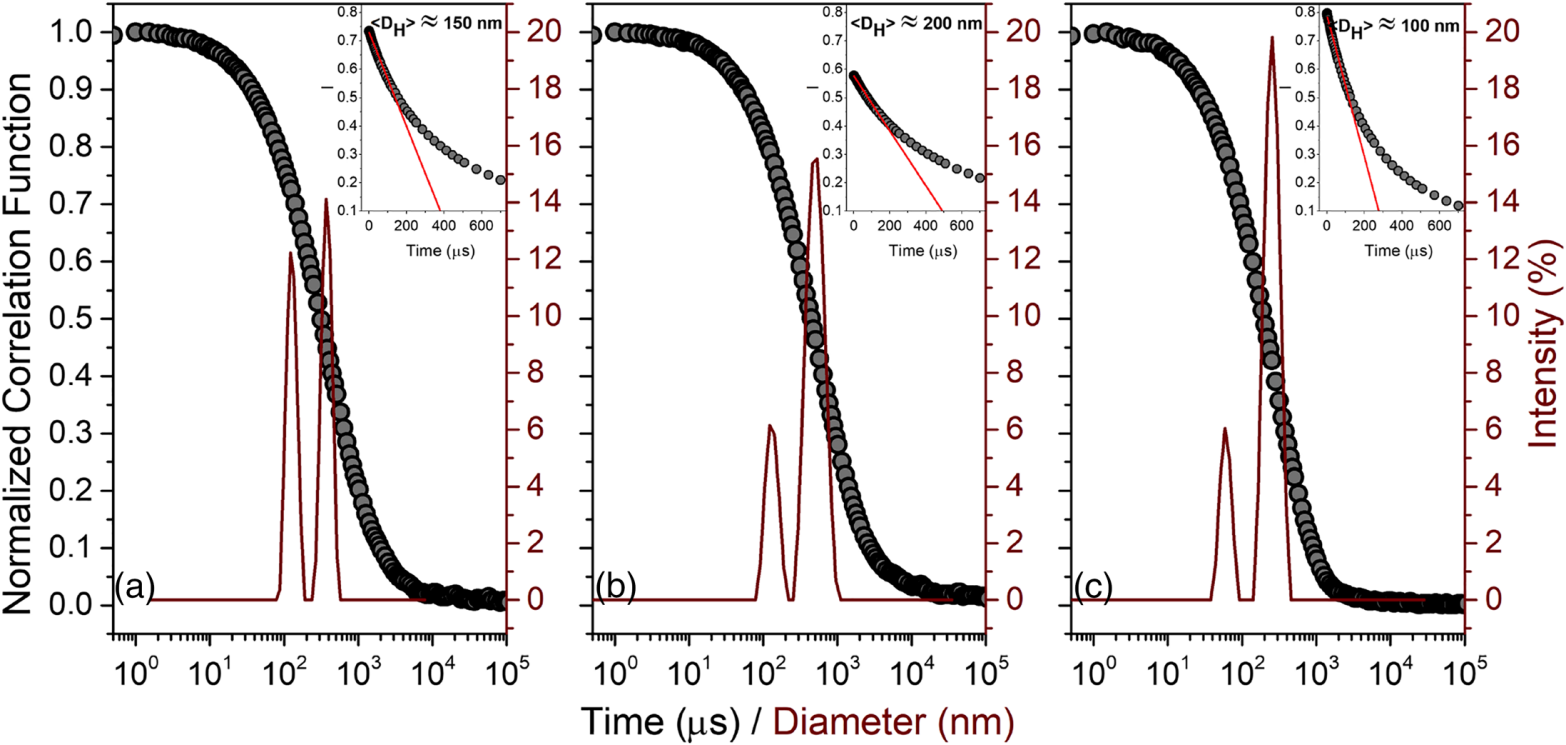
Correlation function and size distribution calculated by means of multiple narrow fit algorithm for water from River Lyckeby (a), River Mörrum (b), and River Helge (c). The inset shows the first slope fitting of the correlation function and the relative averaged diameter that matches with the average between the two distributions coming from the algorithm.

The average size of aggregates in the river waters was obtained from the initial slope of the correlation function from the DLS measurements (Figure 3 insets). The average diameter of the aggregates was 100, 150, and 200 nm for Rivers Helge, Lyckeby, and Mörrum, respectively. In unfiltered river samples of all three rivers, two main size distributions were found, as demonstrated by correlation functions and the relative size distributions obtained by the Multiple Narrow Fit algorithm (Figure 3). River Helge exhibited size distributions between 20–70 and 150–400 nm, while Rivers Lyckeby and Mörrum exhibited distributions around 100–200 and 300–900 nm. It should be noted that these results arise from wide size distributions and unknown aggregate morphology, as well as interference between translational and internal motions of the aggregate clusters displaying a variation in size. As a result, with the methods applied in this study, the size distributions describe a range. The intensity distribution is weighted according to the scattering intensity of each particle fraction. The intensity‐based result can, therefore, be highly sensitive to very small numbers of aggregates, as scattering intensity is proportional to the 6th power of the particle radius. Consequently, the obtained size distributions can be used to distinguish between different size aggregates but do not reflect the number of aggregates within the same size fraction (Berne & Pecora, 2000). Thus, the analyses reflect a wide and continuous range of sizes, and the actual distributions obtained are a product of how the data are analyzed. Still the models provide a useful approach to describe the overall colloidal properties and the relative difference between samples, but the distributions should not be viewed as distinctly different groups of molecules. Moreover, the colloidal characteristics of river waters are most likely not in equilibrium but represent a dynamic system.

Pokrovsky and Schott (2002) detected Fe‐rich colloids in the size range of 0.22–0.8 μm, which matches the size distribution in the range of 300–900 nm observed in the present study. The two size distributions detected in River Helge were similar to those identified in river water by small‐angle neutron scattering (SANS), where the two largest distributions were 20–50 and 50–200 nm (Jarvie & King, 2007). King and Jarvie (2012) attributed the largest of these distributions to the presence of interparticle bridging between the primary particles. SANS contrast matching identified smaller clusters of higher fractal dimension when focusing on the organic component while larger clusters of lower fractal dimension were observed when the contrast conditions favored the mineral component (King & Jarvie, 2012). A bimodal aggregate distribution for river waters has also been proposed from flow field‐flow fractionation (FlFFF) results (Lyvén et al., 2003; Stolpe & Hassellöv, 2007). The FlFFF technique only detects colloids <50 nm and has identified a small (2–3 nm) carbon‐rich phase and a larger (>3 nm) Fe‐rich phase (Lyvén et al., 2003; Stolpe & Hassellöv, 2007). The latter matched with the distribution detected herein for the River Helge, while the smallest fraction was not observed in the unfiltered samples. Probably the scattering intensity from these unfiltered samples was dominated by larger particles where the correlation of the signal takes a long time to decay, and as a consequence small colloids were not detectable.

### 3.2 Filtration Experiments

Filtration through 0.45‐, 0.2‐, and 0.1‐μm filters removed a substantial amount of the Fe from all river waters (Table 1). In River Mörrum, as much as 62% of Fe was lost, independent of filter pore size. In Rivers Helge and Lyckeby, approximately 25% of the Fe was lost by 0.45‐μm filtration and slightly more was removed with decreasing pore size. This goes along with previous studies suggesting that a large proportion of riverine Fe is present in the >0.22‐μm fraction (Hirst et al., 2017; Pokrovsky & Schott, 2002). In sharp contrast, only around 5% of OC was removed by filtration, independent of filter pore size and river water (Table 1). This is in agreement with previous studies showing a major loss of Fe during sequential filtration (5, 0.8, and 0.22 μm: 100, 10, and 1 kDa) of lake and river waters from NW Russia, while most OC was unaffected even by ultrafiltration (Pokrovsky & Schott, 2002).

**Figure 4:**
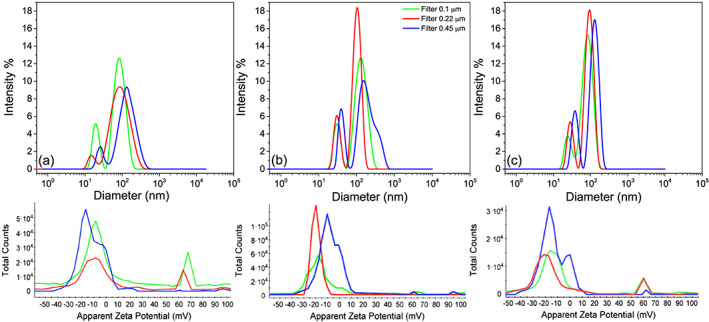
Size distributions of water from River Lyckeby (a), River Mörrum (b), and River Helge (c) after filtration with 0.1‐, 0.22‐, and 0.45‐μm filters. The lower panels show the corresponding zeta potential distributions.

DLS detected two size distributions in all filtered samples (Figure 4). Clearly, when the largest aggregates—from here on referred to as the large distribution—were removed and no longer dominated the scattering, a smaller distribution between 10 and 40 nm could be detected. The second distribution, centered between 100 and 200 nm, corresponds to the smaller distribution detected in unfiltered samples (Figure 3) and hereafter referred to as the intermediate distribution. The presence of two distributions in the filtered samples was supported by measurements of the zeta potential. In samples filtered through 0.1 and 0.22 μm, both negatively (−13 mV) and positively (+60 mV) charged particles were observed. The fact that the relative signal intensity of the positively charged particles increases after filtration through the smaller pore size showed that these correspond to the smallest size distribution. In the presence of intermediate and larger colloids in unfiltered samples, our techniques were unable to detect these small positively charged particles, as discussed above. The coexistence of positively and negatively charged colloids in freshwaters may seem unexpected. However, the negatively charged species were only weakly charged (zeta potential ≈ −10 mV), which suggests negligible interactions with the positively charged nanoparticles.

The smallest distribution detected in filtered waters of all rivers was centered at ~18 nm and displayed a zeta potential between +60 and +70 mV. These small colloids were of similar size as the Fe‐rich colloids detected in river water by FlFFF (Lyvén et al., 2003; Stolpe et al., 2005). Given the circumneutral or slightly acidic pH of the river waters and the relatively high point of zero charge (PZC) of all common iron oxides (Schwertmann & Cornell, 2008), this size fraction was likely attributed to Fe(oxy)hydroxide nanoparticles. Depending on the degree of the surface coverage, the positive charge of these nanoparticles can be neutralized or even reversed, which is fundamental for the colloidal stability in suspension (Hassellov & von der Kammer, 2008). Indeed, depending on the chemical conditions, in particular the concentration of surface reactive anions, both positively and negatively charged Fe(oxy)hydroxide nanoparticles have been observed at circumneutral pH values (Baalousha, 2009; Chekli, Phuntsho, Roy, Lombi, et al., 2013; Cromières et al., 2002). Moreover, Fe(oxy)hydroxide colloids in a similar size range, and without interaction with OM, were identified in River Lena by transmission electron microscopy (TEM) and scanning transmission X‐ray microscopy (STXM) (Hirst et al., 2017). It should be pointed out that we cannot exclude that the occurrence of positively charged nanoparticles originates from bigger aggregates of Fe(oxy)hydroxides fragmented by filtration or centrifugation; however, based on experimental evidence, disintegration is unlikely to occur (section 3.3).

The colloids in the intermediate size range 100–200 nm displayed a distinctly different zeta potential between −12 and −20 mV. The negative zeta potential was likely caused by OM containing carboxylic functional groups (Dickson et al., 2012; Hajdú et al., 2009; Hu et al., 2010). Previous XAS studies have demonstrated that Fe(III) form complexes involving carboxylic functional groups (Karlsson & Persson, 2012). The detection of an organic‐rich carrier phase with an intermediate size distribution (100–200 nm) is in contrast to previous FIFFF studies where organic colloids were assigned much smaller sizes between 0.5 and 3 nm (Hassellov et al., 2007; Krachler et al., 2012; Stolpe & Hassellöv, 2007). FlFFF has much higher size resolution (Lead et al., 1999), and it is possible that larger aggregates dominating the scattering intensity in our DLS measurements prevented the detection of the smaller organic colloids. Moreover, FIFFF analysis only consider size fractions <50 nm.

The largest size distribution that was retained on the filters was high in Fe and low in OC (Table 1), suggesting that it was dominated by Fe(oxy)hydroxides. The significant loss of Fe by filtration demonstrated that a large fraction of Fe was present as large‐sized colloids in agreement with Pokrovsky and Schott (2002). The formation of large clusters of Fe(oxy)hydroxides induced by OM has been demonstrated using cryo‐TEM and scattering experiments in ferrihydrite‐soil OM system (Gentile et al., 2018). Moreover, the fact that absorbance at 420 nm was significantly reduced despite the low retention of OC suggested that the OC retained was highly chromophoric. Krachler et al. (2010) showed that most Fe is indeed bound to high molecular weight chromophoric OM.

The positively charged nanoparticles (10–40 nm) in the smallest size distribution as well as the largest Fe‐rich size distribution (300–900 nm) identified by DLS were likely both dominated by Fe(oxy)hydroxides and contributed to the Fe(oxy)hydroxide phase detected by means of XAS. Consequently, the Fe‐OM complexes also identified by XAS were likely present mainly in the intermediate size distribution with a negative zeta potential. However, the presence of Fe(oxy)hydroxide colloids associated with OM in the intermediate fraction cannot be ruled out.

### 3.3 Salinity Experiments

**Figure 5:**
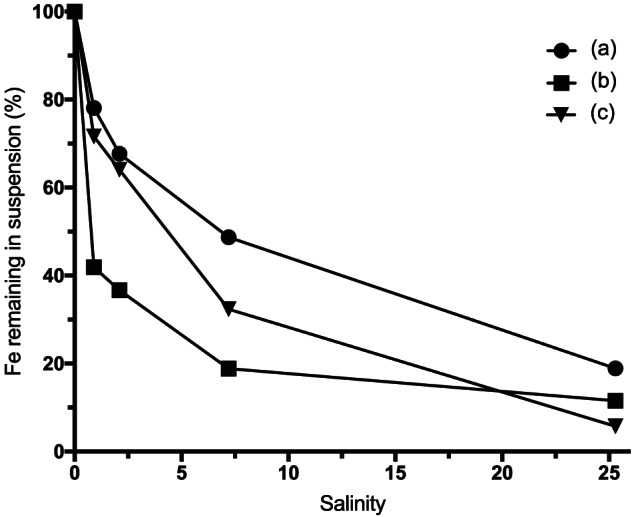
Percentage of Fe remaining in suspension at increasing salinity of River Lyckeby (A), River Mörrum (B), and River Helge (C).

A major loss of Fe in suspension was observed with increasing salinity (Figure 5). OC concentration on the other hand was little affected by increasing salinity, which is in agreement with previous studies on the same and similar systems (Kritzberg et al., 2014; Oʼday et al., 2004; Shiller & Boyle, 1991). Salinity‐induced aggregation and subsequent loss of Fe was lowest for water River Lyckeby (81%), which showed the highest contribution of Fe‐OM complexes (Figure 1 and Table 2). Fe from Rivers Mörrum and Helge aggregated at lower salinities, and 89% and 94% were lost at salinity 25. This is in line with previous studies showing that Fe transport capacity is influenced by the Fe speciation; that is, Fe stability is positively correlated with the relative contribution of Fe‐OM complexes to the total Fe (Herzog et al., 2017; Krachler et al., 2012). Furthermore, the salinity‐induced aggregates from River Lyckeby were strongly dominated by Fe(oxy)hydroxides (Figure 1d and Table 2). Given the relatively high contribution of Fe‐OM complexes to the water from River Lyckeby, this corroborates previous observations of selective removal of Fe(oxy)hydroxides in response to increasing salinity (Herzog et al., 2017). While studies have shown that loss of total Fe in artificial salinity additions compare well to declines in total Fe concentrations along estuarine salinity gradients (Herzog et al., 2020), methodological limitations hinder the determination of Fe speciation of in situ marine samples. However, complementary techniques such as cathodic stripping voltammetry (CSV) have shown the importance of ligand complexation for keeping Fe in suspension in saline waters (Gledhill & Buck, 2012; Laglera et al., 2011).

The X‐ray absorption near edge structure (XANES) data for the aggregated sample from River Lyckeby were similar to the water samples from Rivers Helge and Mörrum, although the double peak in the first derivative was more distinct, indicating an even higher contribution of Fe(oxy)hydroxides (Figure 2). The low contribution of Fe‐OM complexes in the aggregates suggests that the chelate formation indicated by the Fe‐C path contributes to the stability of the Fe at higher salinity (Blazevic et al., 2016; Herzog et al., 2017). Aggregation is caused by weakening of repulsive forces as a result of a compression of the diffuse double layer of the colloids. This allows van der Waals' forces to connect the colloids and the formation of larger aggregates (Hassellov & von der Kammer, 2008). The high contribution of OM in Fe‐OM colloids may supply enough negative charge to remain dispersed even at higher ionic strength (Hassellov & von der Kammer, 2008). The loss of Fe at low salinities was more pronounced in water from River Mörrum. The XAS analyses suggest Fe speciation in Rivers Mörrum and Helge to be similar, but the colloidal aggregates were on average larger in River Mörrum (Figure 3).

The stability of charged colloids is often analyzed within the Derjaguin‐Landau‐Verwey‐Overbeek (DLVO) theory (Evans & Wennerström, 1999). Excess electrolyte screens the long‐range electrostatic repulsion that may lead to particle aggregation, driven by the attractive van der Waals' force. The critical coagulation concentration (CCC) (Evans & Wennerström, 1999) is defined as the electrolyte concentration above which there is no longer a repulsive barrier preventing aggregation, and the colloids aggregate fast with diffusion limited kinetics. However, aggregation can occur already for electrolyte concentrations that are an order of magnitude lower than CCC (Holthoff et al., 1996), although with a reduced rate that decreases with decreasing electrolyte concentration (Evans & Wennerström, 1999). Within the DLVO theory, the CCC is given by (Evans & Wennerström, 1999)

(2) $$\mathrm{CCC}\approx \frac{100{\left(\varepsilon_{0}\varepsilon_{r}\right)}^{3}{\left(k_{B}T\right)}^{5}}{{\left(\mathrm{ze}\right)}^{6}HN_{A}}\Gamma_{0}^{4}$$

in molar concentration, where Γ_0_ is given by

(3) $$\Gamma_{0}=\tanh\frac{\mathrm{ze}\psi_{0}}{4k_{B}T}.$$

Here *ε*_0_ = 8.85 10^−12^ F m^−1^ is the permittivity of vacuum, *ε*_r_ = 78 is the solvent (water) dielectric constant, *k*_*B*_ = 1.38 10^−23^ J is Boltzmann's constant, *T* is the absolute temperature, *e* = 1.6 10^−19^ C is the elementary charge, *z* is the counter ion valency, *H* is the Hamaker constant, *N*_*A*_ = 6.0 10^23^ is Avogadro's number, and *ψ*_0_ is the electrostatic particle surface potential. For iron oxide particles in water, *H* has a value of ca. 3.5 10^−20^ J (Faure et al., 2011).

As seen in Figure 5, we observed a significant precipitation of Fe already at salinity 1 g L^−1^ and the precipitation increased with increasing salinity. These results are consistent with the measured zeta potential of ca. 60 mV. We can estimate a CCC using equations (2) and (3). Approximating the surface potential to be equal to the zeta potential, and setting *z* = 1 (Cl^−^ is the main anion in sea water) and *T* = 293 K, we obtain CCC ≈ 10 g L^−1^ (NaCl). Thus, above this salinity we expect a rapid coagulation and precipitation of the particles, while (slow) aggregation may occur already at salinity 1. We note also that a CCC value of 0.5 g L^−1^ NaCl has been reported for Fe(OH)_3_ colloidal particles (Hunter, 1999).

**Figure 6:**
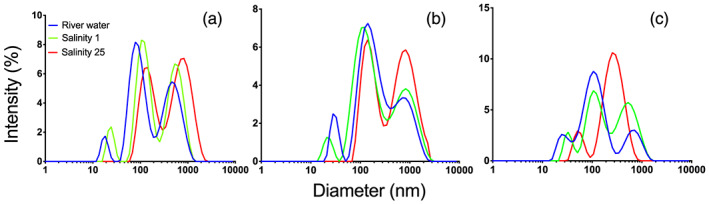
Size distributions after centrifugation of river water at salinity 0, 1, and 25 from River Lyckeby (a), River Mörrum (b), and River Helge (c).

Centrifugation of river water resulted in a removal of larger aggregates, so that the smallest size distribution (10–40 nm) was detected (Figure S1), similar to the filtration experiment. From the fact that size distributions after centrifugation and filtration were comparable, it seems unlikely that the smallest distribution is the result of disintegration of larger colloids. Centrifugation did not entirely remove the largest distribution, resulting in three detectable size distributions as seen in Figure 6 (see Figure S2 for salinity 2 and 7). After increasing the salinity to 25 the smallest size distribution was no longer detected in any of the three river waters. Thus, the increasing salinity induced aggregation of the smallest size distribution, and this selective removal of Fe(oxy)hydroxide nanoparticles was in line with previous studies (Herzog et al., 2017; Krachler et al., 2012; Stolpe & Hassellöv, 2007). The intermediate and large size distributions, which we propose were associated with OM, were still detected at high salinity. While no quantitative conclusions can be drawn regarding the response of these two distributions, it is notable that such large particles remained in suspension despite high salinity. This suggests that these large colloids were not charge stabilized; that is, they did not require long‐range electrostatic repulsion to avoid aggregation due to van der Waals' interactions.

**Figure 7:**
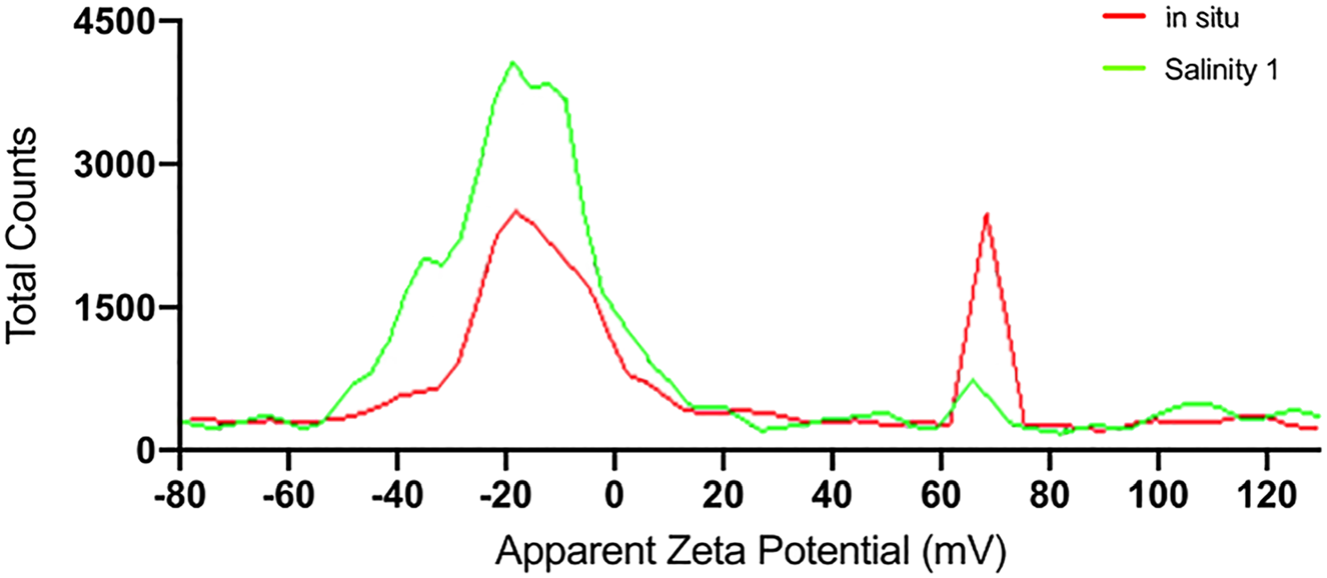
Zeta potential after filtration with 0.1 μm of river water and at salinity 1 of River Lyckeby.

From Figure 6 it appears that the effect on colloidal distribution was minor at salinity 1. However, to facilitate better resolution of how salinity affects the size distribution, filtered (0.1‐μm) water from River Lyckeby was compared to filtered water amended with salt solution to salinity 1. Already at this low salinity a reduction of positively charged colloids was detected (Figure 7). This is in line with the selective removal of the Fe(oxy)hydroxides as suggested by the aggregation experiments (Figures 1a and 1d). Moreover, it shows that the selective removal of Fe starts already at low salinity. In many previous studies the large majority of riverine Fe was lost from suspension in the low salinity range, whereas in some systems maximum removal occurred at higher salinity (Herzog et al., 2020, and Lyckeby and Helge in this study). Possibly, this difference is related to the contribution of nanoparticulate Fe(oxy)hydroxides, but since the information derived from DLS measurements were not quantitative, this cannot be assessed from the current study.

## 4 Conclusions

Given the importance of riverine Fe and OC as carriers of major elements and contaminants, their occurrence, characteristics, and fate across estuarine salinity gradients have been the subject of numerous studies (Bauer et al., 2017; Muller, 2018; Stolpe & Hassellöv, 2007). Many of these studies focused on the smaller size distributions, for example, the filterable fraction (<0.45 μm), to be able to separate supposedly chemically reactive and biologically available Fe from that bound in biomass or in detrital minerals, which may otherwise dominate. The size range studied may also be constrained by the analytical methods applied for characterization. The overarching aim of the current study—to study how riverine Fe and OC survives estuarine mixing and what are the controlling factors—motivated the inclusion of a wider size range. In these boreal rivers, Fe concentrations are exceptionally high, and plankton biomass and detrital minerals make only a minor contribution to total Fe and OC (Herzog et al., 2017). Nevertheless, filtration through 0.45‐μm filters removed between 23% and 62% of Fe. This implies that studies of only the filterable fraction may miss a large fraction of Fe and be less representative of natural conditions. Since particulate, colloidal, and dissolved phases interact and influence the response to increasing salinity (Forsgren et al., 1996), including all size fractions should yield a more comprehensive understanding.

XAS confirmed the contribution of two Fe phases in the river waters—Fe‐OM complexes and Fe(oxy)hydroxides—and three size distributions were identified from DLS measurements. The smallest size distribution (10–40 nm) was positively charged, suggesting the presence of Fe(oxy)hydroxide nanoparticles that were not neutralized by surface interactions with OM (Hirst et al., 2017). This distribution matches the larger Fe‐rich distribution identified by FIFFF in several studies (3–50 nm) (Andersson et al., 2006; Lyvén et al., 2003). The largest distribution identified in our study (300–900 nm) was lost upon filtration (0.1, 0.2, and 0.45 μm). Given the low OC:Fe ratio of particles retained on filters, as well as the drastic loss of color, we inferred that this fraction was dominated by Fe(oxy)hydroxides associated with chromophoric molecular matter. The negative surface charge of the intermediate size distribution (100–200 nm), and the small contribution of OM in the other distributions, suggests that OM colloids were dominating this fraction. Moreover, since XAS identified significant contributions of Fe‐OM complexes, and the smallest and largest distributions were dominated by Fe(oxy)hydroxides, these Fe‐OM complexes are likely associated with the intermediate size distribution.

As for the response to increasing salinity, DLS measurements showed a loss of the smallest distribution at high salinity. Moreover, the signal of the positively charged colloids decreased with increasing salinity, supporting the assumption of a preferential removal of the Fe(oxy)hydroxides. This was corroborated by XAS, which verified selective loss of Fe(oxy)hydroxides, and also by previous FIFFF studies that have demonstrated the loss of Fe‐rich nanocolloids at increasing salinity (Krachler et al., 2012; Stolpe & Hassellöv, 2007). The XAS analyses further showed, in line with previous studies (Herzog et al., 2020), that a higher contribution of Fe‐OM complexes yielded a higher Fe stability with respect to increasing salinities. Although the DLS is not quantitative, the analyses clearly showed that the intermediate size distribution was not lost in response to high salinity. Interestingly, also the largest size distribution, supposedly dominated by Fe(oxy)hydroxides in association with OM, was detected at high salinity.

## Supporting information



Supporting Information S1

## References

[jgrg21629-bib-0001] Andersson, K. , Dahlqvist, R. , Turner, D. , Stolpe, B. , Larsson, T. , Ingri, J. , & Andersson, P. (2006). Colloidal rare earth elements in a boreal river: Changing sources and distributions during the spring flood. Geochimica et Cosmochimica Acta, 70(13), 3261–3274. 10.1016/j.gca.2006.04.021

[jgrg21629-bib-0002] Baalousha, M. (2009). Aggregation and disaggregation of iron oxide nanoparticles: Influence of particle concentration, pH and natural organic matter. Science of the Total Environment, 407(6), 2093–2101. 10.1016/j.scitotenv.2008.11.022 19059631

[jgrg21629-bib-0003] Bauer, S. , Blomqvist, S. , & Ingri, J. (2017). Distribution of dissolved and suspended particulate molybdenum, vanadium, and tungsten in the Baltic Sea. Marine Chemistry, 196, 135–147.

[jgrg21629-bib-0004] Berne, B. J. , & Pecora, R. (2000). Dynamic light scattering: With applications to chemistry, biology, and physics. 2000 (Vol. 376). Mineola, NY: Dover Publications.

[jgrg21629-bib-0005] Blazevic, A. , Orlowska, E. , Kandioller, W. , Jirsa, F. , Keppler, B. K. , Tafili‐Kryeziu, M. , Linert, W. , Krachler, R. F. , Krachler, R. , & Rompel, A. (2016). Photoreduction of terrigenous Fe‐humic substances leads to bioavailable iron in oceans. Angewandte Chemie, 128(22), 6527–6532. 10.1002/ange.201600852 27478277 PMC4949668

[jgrg21629-bib-0006] Boyle, E. A. , Edmond, J. M. , & Sholkovitz, E. R. (1977). The mechanism of iron removal in estuaries. Geochimica et Cosmochimica Acta, 41(9), 1313–1324.

[jgrg21629-bib-0007] Chekli, L. , Phuntsho, S. , Roy, M. , Lombi, E. , Donner, E. , & Shon, H. K. (2013). Assessing the aggregation behaviour of iron oxide nanoparticles under relevant environmental conditions using a multi‐method approach. Water Research, 47(13), 4585–4599. 10.1016/j.watres.2013.04.029 23764608

[jgrg21629-bib-0008] Chekli, L. , Phuntsho, S. , Roy, M. , & Shon, H. K. (2013). Characterisation of Fe‐oxide nanoparticles coated with humic acid and Suwannee River natural organic matter. Science of the Total Environment, 461‐462, 19–27. 10.1016/j.scitotenv.2013.04.083 23712112

[jgrg21629-bib-0009] Cromières, L. , Moulin, V. , Fourest, B. , & Giffaut, E. (2002). Physico‐chemical characterization of the colloidal hematite/water interface: Experimentation and modelling. Colloids and Surfaces A: Physicochemical and Engineering Aspects, 202(1), 101–115. 10.1016/S0927-7757(01)01065-2

[jgrg21629-bib-0010] Dahlqvist, R. , Benedetti, M. F. , Andersson, K. , Turner, D. , Larsson, T. , Stolpe, B. , & Ingri, J. (2004). Association of calcium with colloidal particles and speciation of calcium in the Kalix and Amazon rivers. Geochimica et Cosmochimica Acta, 68(20), 4059–4075. 10.1016/j.gca.2004.04.007

[jgrg21629-bib-0011] Dickson, D. , Liu, G. , Li, C. , Tachiev, G. , & Cai, Y. (2012). Dispersion and stability of bare hematite nanoparticles: Effect of dispersion tools, nanoparticle concentration, humic acid and ionic strength. Science of the Total Environment, 419, 170–177. 10.1016/j.scitotenv.2012.01.012 PMC4696403 22289174

[jgrg21629-bib-0012] Ekström, S. M. , Regnell, O. , Reader, H. E. , Nilsson, P. A. , Löfgren, S. , & Kritzberg, E. S. (2016). Increasing concentrations of iron in surface waters as a consequence of reducing conditions in the catchment area. Journal of Geophysical Research: Biogeosciences, 121, 479–493. 10.1002/2015JG003141

[jgrg21629-bib-0013] Evans, D. F. , & Wennerstrom, H. (1999). Colloidal domain. New York: Wiley‐Vch.

[jgrg21629-bib-0014] Faure, B. , Salazar‐Alvarez, G. , & Bergström, L. (2011). Hamaker constants of iron oxide nanoparticles. Langmuir, 27(14), 8659–8664. 10.1021/la201387d 21644514

[jgrg21629-bib-0015] Filella, M. , Buffle, J. , & Leppard, G. G. (1993). Characterization of submicrometre colloids in freshwaters: Evidence for their bridging by organic structures. Water Science and Technology, 27(11), 91.

[jgrg21629-bib-0016] Forsgren, G. , Jansson, M. , & Nilsson, P. (1996). Aggregation and sedimentation of iron, phosphorus and organic carbon in experimental mixtures of freshwater and estuarine water. Estuarine, Coastal and Shelf Science, 43(2), 259–268.

[jgrg21629-bib-0017] Funke, H. , Scheinost, A. C. , & Chukalina, M. (2005). Wavelet analysis of extended x‐ray absorption fine structure data. Physical Review B, 71(9), 094110.

[jgrg21629-bib-0018] Gentile, L. , Wang, T. , Tunlid, A. , Olsson, U. , & Persson, P. (2018). Ferrihydrite nanoparticle aggregation induced by dissolved organic matter. The Journal of Physical Chemistry A., 122(38), 7730–7738. 10.1021/acs.jpca.8b05622 30165023

[jgrg21629-bib-0019] Gledhill, M. , & Buck, K. N. (2012). The organic complexation of iron in the marine environment: A review. Frontiers in Microbiology, 3, 69. 22403574 10.3389/fmicb.2012.00069 PMC3289268

[jgrg21629-bib-0020] Hajdú, A. , Illés, E. , Tombácz, E. , & Borbáth, I. (2009). Surface charging, polyanionic coating and colloid stability of magnetite nanoparticles. Colloids and Surfaces A: Physicochemical and Engineering Aspects, 347(1–3), 104–108. 10.1016/j.colsurfa.2008.12.039

[jgrg21629-bib-0021] Hassellov, M. , & von der Kammer, F. (2008). Iron oxides as geochemical nanovectors for metal transport in soil‐river systems. Elements, 4(6), 401–406.

[jgrg21629-bib-0022] Hassellov, M. , von der Kammer, F. , & Beckett, R. (2007). Characterisation of aquatic colloids and macromolecules by field‐flow fractionation. IUPAC Series on Analytical and Physical Chemistry of Environmental Systems, 10, 223.

[jgrg21629-bib-0023] Herzog, S. D. , Persson, P. , & Kritzberg, E. S. (2017). Salinity effects on iron speciation in boreal river waters. Environmental Science & Technology, 51(17), 9747–9755. 10.1021/acs.est.7b02309 28836428

[jgrg21629-bib-0024] Herzog, S. D. , Persson, P. , Kvashnina, K. , & Kritzberg, E. S. (2020). Organic iron complexes enhance iron transport capacity along estuarine salinity gradients of Baltic estuaries. Biogeosciences, 17(2), 331–344. 10.5194/bg-17-331-2020

[jgrg21629-bib-0025] Hirst, C. , Andersson, P. S. , Shaw, S. , Burke, I. T. , Kutscher, L. , Murphy, M. J. , Maximov, T. , Pokrovsky, O. S. , Mörth, C.‐M. , & Porcelli, D. (2017). Characterisation of Fe‐bearing particles and colloids in the Lena River basin, NE Russia. Geochimica et Cosmochimica Acta, 213, 553–573. 10.1016/j.gca.2017.07.012

[jgrg21629-bib-0026] Holthoff, H. , Egelhaaf, S. U. , Borkovec, M. , Schurtenberger, P. , & Sticher, H. (1996). Coagulation rate measurements of colloidal particles by simultaneous static and dynamic light scattering. Langmuir, 12(23), 5541–5549. 10.1021/la960326e

[jgrg21629-bib-0027] Hu, J.‐D. , Zevi, Y. , Kou, X. M. , Xiao, J. , Wang, X. J. , & Jin, Y. (2010). Effect of dissolved organic matter on the stability of magnetite nanoparticles under different pH and ionic strength conditions. Science of the Total Environment, 408(16), 3477–3489. 10.1016/j.scitotenv.2010.03.033 20421125

[jgrg21629-bib-0028] Hunter, J. (1999). Introduction to modern colloid science. New York, USA: Oxford University Press.

[jgrg21629-bib-0029] Ingri, J. , Widerlund, A. , Land, M. , Gustafsson, Ö. , Andersson, P. , & Öhlander, B. (2000). Temporal variations in the fractionation of the rare earth elements in a boreal river; the role of colloidal particles. Chemical Geology, 166(1‐2), 23–45. 10.1016/S0009-2541(99)00178-3

[jgrg21629-bib-0030] Jarvie, H. P. , & King, S. M. (2007). Small‐angle neutron scattering study of natural aquatic nanocolloids. Environmental Science & Technology, 41(8), 2868–2873. 10.1021/es061912p 17533851

[jgrg21629-bib-0031] Karlsson, T. , & Persson, P. (2010). Coordination chemistry and hydrolysis of Fe(III) in a peat humic acid studied by X‐ray absorption spectroscopy. Geochimica et Cosmochimica Acta, 74(1), 30–40.

[jgrg21629-bib-0032] Karlsson, T. , & Persson, P. (2012). Complexes with aquatic organic matter suppress hydrolysis and precipitation of Fe(III). Chemical Geology, 322–323, 19–27.

[jgrg21629-bib-0033] Kester, D. R. , Duedall, I. W. , Connors, D. N. , & Pytkowicz, R. M. (1967). Preparation of artificial seawater. Limnology and Oceanography, 12(1), 176–179. 10.4319/lo.1967.12.1.0176

[jgrg21629-bib-0034] King, S. M. , & Jarvie, H. P. (2012). Exploring how organic matter controls structural transformations in natural aquatic nanocolloidal dispersions. Environmental Science & Technology, 46(13), 6959–6967. 10.1021/es2034087 22260303

[jgrg21629-bib-0035] Klementev, K. V. (2001). Statistical evaluations in fitting problems. Journal of Synchrotron Radiation, 8(2), 270–272. 11512749 10.1107/s0909049500015351

[jgrg21629-bib-0036] Klementiev, K. V. (2002). XANES dactyloscope for Windows. KV Klementiev, XANES dactyloscope for Windows, freeware: www.desy.de/~klmn/xanda.html

[jgrg21629-bib-0037] Krachler, R. , Krachler, R. F. , von der Kammer, F. , Süphandag, A. , Jirsa, F. , Ayromlou, S. , Hofmann, T. , & Keppler, B. K. (2010). Relevance of peat‐draining rivers for the riverine input of dissolved iron into the ocean. Science of the Total Environment, 408(11), 2402–2408. 10.1016/j.scitotenv.2010.02.018 20206963

[jgrg21629-bib-0038] Krachler, R. , Kammer, F. , Jirsa, F. , Süphandag, A. , Krachler, R. F. , Plessl, C. , Vogt, M. , Keppler, B. K. , & Hofmann, T. (2012). Nanoscale lignin particles as sources of dissolved iron to the ocean. Global Biogeochemical Cycles, 26, GB3024. 10.1029/2012GB004294

[jgrg21629-bib-0039] Kritzberg, E. S. , Bedmar Villanueva, A. , Jung, M. , & Reader, H. E. (2014). Importance of boreal rivers in providing iron to marine waters. PLoS ONE, 9(9), e107500. 10.1371/journal.pone.0107500 25233197 PMC4169402

[jgrg21629-bib-0040] Laglera, L. M. , Battaglia, G. , & van den Berg, C. M. G. (2011). Effect of humic substances on the iron speciation in natural waters by CLE/CSV. Marine Chemistry, 127(1), 134–143.

[jgrg21629-bib-0041] Lead, J. R. , Hamilton‐Taylor, J. , Davison, W. , & Harper, M. (1999). Trace metal sorption by natural particles and coarse colloids. Geochimica et Cosmochimica Acta, 63(11–12), 1661–1670. 10.1016/S0016-7037(99)00006-X

[jgrg21629-bib-0042] Lead, J. R. , & Wilkinson, K. J. (2006). Aquatic colloids and nanoparticles: Current knowledge and future trends. Environmental Chemistry, 3(3), 159–171.

[jgrg21629-bib-0043] Lyvén, B. , Hassellöv, M. , Turner, D. R. , Haraldsson, C. , & Andersson, K. (2003). Competition between iron‐and carbon‐based colloidal carriers for trace metals in a freshwater assessed using flow field‐flow fractionation coupled to ICPMS. Geochimica et Cosmochimica Acta, 67(20), 3791–3802. 10.1016/S0016-7037(03)00087-5

[jgrg21629-bib-0044] Maillot, F. , Morin, G. , Wang, Y. , Bonnin, D. , Ildefonse, P. , Chaneac, C. , & Calas, G. (2011). New insight into the structure of nanocrystalline ferrihydrite: EXAFS evidence for tetrahedrally coordinated iron (III). Geochimica et Cosmochimica Acta, 75(10), 2708–2720. 10.1016/j.gca.2011.03.011

[jgrg21629-bib-0045] Muller, F. L. L. (2018). Exploring the potential role of terrestrially derived humic substances in the marine biogeochemistry of iron. Frontiers in Earth Science, 6, 159.

[jgrg21629-bib-0046] Neubauer, E. , Köhler, S. J. , von der Kammer, F. , Laudon, H. , & Hofmann, T. (2013). Effect of pH and stream order on iron and arsenic speciation in boreal catchments. Environmental Science & Technology, 47(13), 7120–7128. 10.1021/es401193j 23692297

[jgrg21629-bib-0047] Oʼday, P. A. , Nelson Rivera, R. R. Jr. , & Carroll, S. A. (2004). X‐ray absorption spectroscopic study of Fe reference compounds for the analysis of natural sediments. American Mineralogist, 89(4), 572–585. 10.2138/am-2004-0412

[jgrg21629-bib-0048] Oni, S. K. , Futter, M. N. , Bishop, K. , Köhler, S. J. , Ottosson‐Löfvenius, M. , & Laudon, H. (2013). Long‐term patterns in dissolved organic carbon, major elements and trace metals in boreal headwater catchments: Trends, mechanisms and heterogeneity. Biogeosciences, 10(4), 2315–2330. 10.5194/bg-10-2315-2013

[jgrg21629-bib-0049] Palmer, N. E. , & von Wandruszka, R. (2001). Dynamic light scattering measurements of particle size development in aqueous humic materials. Fresenius' Journal of Analytical Chemistry, 371(7), 951–954. 10.1007/s002160101037 11769806

[jgrg21629-bib-0050] Persson, P. , & Axe, K. (2005). Adsorption of oxalate and malonate at the water‐goethite interface: Molecular surface speciation from IR spectroscopy. Geochimica et Cosmochimica Acta, 69(3), 541–552.

[jgrg21629-bib-0051] Pokrovsky, O. S. , & Schott, J. (2002). Iron colloids/organic matter associated transport of major and trace elements in small boreal rivers and their estuaries (NW Russia). Chemical Geology, 190(1), 141–179.

[jgrg21629-bib-0052] Pokrovsky, O. S. , Schott, J. , & Dupré, B. (2006). Trace element fractionation and transport in boreal rivers and soil porewaters of permafrost‐dominated basaltic terrain in Central Siberia. Geochimica et Cosmochimica Acta, 70(13), 3239–3260.

[jgrg21629-bib-0053] Pokrovsky, O. S. , Shirokova, L. S. , Viers, J. , Gordeev, V. V. , Shevchenko, V. P. , Chupakov, A. V. , Vorobieva, T. Y. , Candaudap, F. , Causserand, C. , Lanzanova, A. , & Zouiten, C. (2014). Fate of colloids during estuarine mixing in the Arctic. Ocean Science, 10(1), 107–125. 10.5194/os-10-107-2014

[jgrg21629-bib-0054] Schwertmann, U. , & Cornell, R. M. (2008). Iron oxides in the laboratory. New York: John Wiley & Sons.

[jgrg21629-bib-0055] Shiller, A. M. , & Boyle, E. A. (1991). Trace elements in the Mississippi River Delta outflow region: Behavior at high discharge. Geochimica et Cosmochimica Acta, 55(11), 3241–3251.

[jgrg21629-bib-0056] Sholkovitz, E. R. , Boyle, E. A. , & Price, N. B. (1978). The removal of dissolved humic acids and iron during estuarine mixing. Earth and Planetary Science Letters, 40(1), 130–136.

[jgrg21629-bib-0057] Sjöstedt, C. , Persson, I. , Hesterberg, D. , Kleja, D. B. , Borg, H. , & Gustafsson, J. P. (2013). Iron speciation in soft‐water lakes and soils as determined by EXAFS spectroscopy and geochemical modelling. Geochimica et Cosmochimica Acta, 105, 172–186. 10.1016/j.gca.2012.11.035

[jgrg21629-bib-0058] Stolpe, B. , & Hassellöv, M. (2007). Changes in size distribution of fresh water nanoscale colloidal matter and associated elements on mixing with seawater. Geochimica et Cosmochimica Acta, 71(13), 3292–3301.

[jgrg21629-bib-0059] Stolpe, B. , & Hassellöv, M. (2010). Nanofibrils and other colloidal biopolymers binding trace elements in coastal seawater: Significance for variations in element size distributions. Limnology and Oceanography, 55(1), 187–202.

[jgrg21629-bib-0060] Stolpe, B. , Hassellöv, M. , Andersson, K. , & Turner, D. R. (2005). High resolution ICPMS as an on‐line detector for flow field‐flow fractionation; multi‐element determination of colloidal size distributions in a natural water sample. Analytica Chimica Acta, 535(1–2), 109–121. 10.1016/j.aca.2004.11.067

[jgrg21629-bib-0061] Stumm, W. , & Morgan, J. J. (2012). Aquatic chemistry: Chemical equilibria and rates in natural waters (Vol. 126). New York: John Wiley & Sons.

[jgrg21629-bib-0062] Sundman, A. , Karlsson, T. , Laudon, H. , & Persson, P. (2014). XAS study of iron speciation in soils and waters from a boreal catchment. Chemical Geology, 364, 93–102. 10.1016/j.chemgeo.2013.11.023

[jgrg21629-bib-0063] Webb, S. M. (2005). SIXpack: A graphical user interface for XAS analysis using IFEFFIT. Physica Scripta, 2005(T115), 1011.

[jgrg21629-bib-0064] Wilke, M. , Farges, F. , Petit, P.‐E. , Brown, G. E. Jr. , & Martin, F. (2001). Oxidation state and coordination of Fe in minerals: An Fe K‐XANES spectroscopic study. American Mineralogist, 86(5–6), 714–730. 10.2138/am-2001-5-612

[jgrg21629-bib-0065] Wojdyr, M. (2010). Fityk: A general‐purpose peak fitting program. Journal of Applied Crystallography, 43(5), 1126–1128.

[jgrg21629-bib-0066] Zabinsky, S. I. , Rehr, J. J. , Ankudinov, A. , Albers, R. C. , & Eller, M. J. (1995). Multiple‐scattering calculations of X‐ray‐absorption spectra. Physical Review B, 52(4), 2995–3009. 10.1103/PhysRevB.52.2995 9981373

