# Supplementary material for: Characterization of Iron and Organic Carbon Colloids in Boreal Rivers and Their Fate at High Salinity
Source: J Geophys Res Biogeosci. 2020 Apr 7;125(4):e2019JG005517. doi: 10.1029/2019JG005517 (PMC8023251; doi:10.1029/2019JG005517)
Supplement: Supplementary file 1 — Supporting Information S1 [file JGRG-125-e2019JG005517-s001.docx]

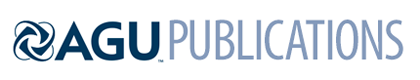


***JGR: Biogeosciences***

**Supporting Information for**

Characterization of Iron and Organic Carbon Colloids in Boreal Rivers and their Fate at high Salinity

Simon David Herzog^1*†^, Luigi Gentile^2*^, Ulf Olsson^3^, Per Persson^4^, and Emma Sofia Kritzberg^1^

^1^Department of Biology/Aquatic Ecology, Lund University, SE-223 62, Lund, Sweden

^2^Department of Biology, MEMEG Unit, Lund University, Lund 223 62, Sweden

^3^Department of Chemistry, Physical Chemistry Division, Lund University, Lund 22362,Sweden

^4^Centre for Environmental and Climate Research & Department of Biology, Lund University, SE-223 62, Lund, Sweden

^1^Department of Biology/Aquatic Ecology, Lund University, SE-223 62, Lund, Sweden

*Shared first authorship

†Corresponding author: Simon David Herzog (simon.herzog@biol.lu.se)

**Contents of this file**

Text S1

Figures S1 to S2

Tables S1 to S2

**Introduction**

The supporting information contains a detailed description of the XAS analysis and results (Text S1). Size distribution before and after centrifugation of river water resulted in a removal of larger aggregates as seen in Figure S1 and S2 for salinity 1, 2 and 7 of all rivers. Table S1 shows the amount of organic matter (OC) removed by filtration and in Table S2 the values of the integrated pre-edge intensity and centroid position are listed showing the predominance of Fe(III) of all samples. The raw data set can be downloaded from the repository Zenodo, doi: 10.5281/zenodo.3707589.

Text S1

XAS results

When quantitatively interpreting the WT plots, the different backscattering atoms appear in different areas in the plot, with heavy elements appearing at higher k-values compared to lighter elements. This allows us to differentiate between Fe (oxy)hydroxide originating from the Fe− Fe scattering paths at higher k-values at ca. 7.5 Å−k, 2.8 Å and the Fe-OM with the Fe-C and Fe-C-C(O or N) paths at lower k-values at ca. 3 Å−1, 2.5 Å , respectively 3 Å−1, 3.2−3.7 Å. The location of the heavy features found in the river samples are in good agreement WT plots from model compounds of ferrihydrite (Sundman et al. 2014) and goethite (Karlsson and Persson 2010), whereas the lighter features in in good agreement with trisoxalatoiron(III) (Karlsson and Persson 2010).

The shell-by-shell fitting of the EXAFS scans contained five scattering paths giving more information on the local coordination environment and further, allowed to distinguish between the two main phases (Table 2). Beyond the first shell (Fe−O), a short and a longer Fe-Fe path were used to model Fe (oxy)hydroxide and a Fe−C path and a Fe−C/O multiple scattering path represented the Fe-OM. The modeling fits corroborated the WT plots and also here the Fe-Fe paths showed similar distances to ferrihydrite (Szytuła et al. 1968). Fe-C distances were close to the one of chelating Fe−OM structures, suggesting the an 6-membered ring or a mixtures between 5- and 6-membered ring structure (Persson and Axe 2005). The coordination number (CN), which indicates the contribution of the different paths in the samples, showed again that all samples contained a substantial proportions of both Fe (oxy)hydroxide and Fe−OM complexes, however with a variation in the relative contribution of the Fe phases. A CN ratio was calculated by dividing the CN od the Fe-C by the CN of the short Fe-Fe, to show the distribution of the two phases in the samples (Table 1). The CN ratio indicates that Lyckeby showed a high contribution of the Fe-OM phases, whereas both Helge and Mörrum showed a more even contribution of both phases.

The position of the pre and main edge for all samples was in agreement with a predominance of Fe(III) (Figure 2 I), which is in good agreement with the literature (Wilke et al. 2001). The oxidation state was further confirmed by the pre-edge centroid energy, which for all samples was close to 7113.7eV indicating solely the occurrence of Fe(III) (Table S2). The predominance of Fe(III) is in agreement to previous studies on river mouth samples (Herzog et al. 2019). Further the integrated pre edge intensity was similar among all samples indicating the presence of 6-coordinated Fe. The double peak detected in the first derivative of the XANES spectra for River Helgeå and Mörrum is an indication for dominance of iron oxides (Figure 2 a tII) and might indicate a contribution from minerogenic Fe (Sundman et al. 2014). For River Lyckeby this feature was less distinct and the presence of a single peak is suggesting a dominance of amorphous Fe (oxy)hydroxides and of Fe-OM (Sundman et al. 2014) and corroborates with the results from the WT plots.

Figure S1

**Figure S1.** Size distribution in nm of river water before (black) and after (blue) centrifugation of River Lyckeby, River Mörrum and River Helge.

Figure S2

Figure S2. Size distribution in nm of River Lyckeby, River Mörrum and River Helge at salinity 1, 2 and 7 after centrifugation.

Table S1

Table S1. OC concentration (mg/l) in river water samples (RW) and filtered river water samples with different filter sizes (0.1, 0.22 and 0.45 μm).

| **River** | ***unfiltered*** | **0.45 μm** | **0.22 μm** | **0.1 μm** |
| --- | --- | --- | --- | --- |
| **R. Lyckeby** | 15.3 | 14.6 | 14.6 | 14.7 |
| **R. Mörrum** | 7.6 | 7.2 | 7.1 | 7.2 |
| **R. Helge** | 9.8 | 9.5 | 9.3 | 9.6 |

Table S2
Table S2. XANES integrated pre-edge intensity and centroid position of all samples.

| **River** | **Integrated pre edge intensity** | **Centroid position (eV)** |
| --- | --- | --- |
| **R. Lyckeby** | 0.0770 | 7113.63 |
| **R. Mörrum** | 0.0541 | 7113.80 |
| R. Helge | 0.0867 | 7113.60 |
| **Lyckeby**  **aggregates** | 0.0849 | 7113.64 |
